# Large-Scale and Highly Efficient Production of Ultrafine PVA Fibers by Electro-Centrifugal Spinning for NH_3_ Adsorption

**DOI:** 10.3390/ma16072903

**Published:** 2023-04-06

**Authors:** Youye Ma, Kanghui Cai, Guojie Xu, Yueling Xie, Peng Huang, Jun Zeng, Ziming Zhu, Jie Luo, Huawen Hu, Kai Zhao, Min Chen, Kun Zheng

**Affiliations:** 1 School of Materials Science and Hydrogen Energy, Foshan University, Foshan 528000, China; ma13415778069@163.com (Y.M.); caikhrun@163.com (K.C.); dalnim1632@163.com (Y.X.); 17802074708@163.com (P.H.); huawenhu@126.com (H.H.); zhaokai@fosu.edu.cn (K.Z.); minchen1981@126.com (M.C.); 2 Guangdong Key Laboratory for Hydrogen Energy Technologies, Foshan University, Foshan 528000, China; 3 China Foshan Nanofiberlabs Co., Ltd., Foshan 528225, China; xuguojie@qingzitech.com (G.X.); fungomail@126.com (J.Z.); sevident_zhu@163.com (Z.Z.); 4 School of Chemistry and Chemical Engineering, Guangxi University, Nanning 530005, China; 5 State Key Laboratory of Precision Electronic Manufacturing Technology and Equipment, Guangdong University of Technology, Guangzhou 510006, China; 6 Department of Hydrogen Energy, Faculty of Energy and Fuels, AGH University of Science and Technology, al. A. Mickiewicza 30, 30-059 Krakow, Poland; 7 AGH Centre of Energy, AGH University of Science and Technology, ul. Czarnowiejska 36, 30-054 Krakow, Poland

**Keywords:** ultrafine fiber, electrostatic-field-assisted centrifugal spinning, polyvinyl alcohol (PVA), highly efficient production, orthogonal experiment

## Abstract

Ultrafine Polyvinyl alcohol (PVA) fibers have an outstanding potential in various applications, especially in absorbing fields. In this manuscript, an electrostatic-field-assisted centrifugal spinning system was designed to improve the production efficiency of ultrafine PVA fibers from PVA aqueous solution for NH_3_ adsorption. It was established that the fiber production efficiency using this self-designed system could be about 1000 times higher over traditional electrospinning system. The produced PVA fibers establish high morphology homogeneity. The impact of processing variables of the constructed spinning system including rotation speed, needle size, liquid feeding rate, and voltage on fiber morphology and diameter was systematically investigated by SEM studies. To acquire homogeneous ultrafine PVA fiber membranes, the orthogonal experiment was also conducted to optimize the spinning process parameters. The impact weight of different studied parameters on the spinning performance was thus provided. The experimental results showed that the morphology of micro/nano-fibers can be well controlled by adjusting the spinning process parameters. Ultrafine PVA fibers with the diameter of 2.55 μm were successfully obtained applying the parameters, including rotation speed (6500 rpm), needle size (0.51 mm), feeding rate (3000 mL h^−1^), and voltage (20 kV). Furthermore, the obtained ultrafine PVA fiber mat was demonstrated to be capable of selectively adsorbing NH_3_ gas relative to CO_2_, thus making it promising for NH_3_ storage and other environmental purification applications.

## 1. Introduction

Micro/nano-fibers have been widely used in a wide range of applications, due to their very promising characteristics of large specific surface area, selective permeability, and surface adsorption properties [1]. As a kind of water-soluble polymer with biodegradability, biocompatibility, and nontoxicity, polyvinyl alcohol (PVA) possesses very good chemical and thermal stability with a broad spectrum of applications [2]. PVA is also a very excellent candidate to fabricate micro/nano-fibers with the highest ultimate strength and modulus in theory. Such a unique property makes PVA adaptive for various spinning methods, such as phase separation [3], self-assembly, template synthesis [4], drawing [5], and electrospinning (ES). The ES method has been widely used because of its advantages including: simple preparation technology, good tenability, and strong technical combination [6]. Although the ES system is considered the most effective and versatile technique for producing fibers with nanoscale diameters, there are evident limitations for such a system. For instance, many polymers applied for electrospinning are dissolved in highly toxic chlorinated or fluorinated solvents, thus making the electrospinning process environmentally unamiable [7,8]. A high electrostatic field is usually required to facilitate the conventional fiber production, thus generating high costs and safety issues [9]. Moreover, the electrospinning process is rather inefficient, with solution feeding rates of as small as 1 mL h^−1^ [10,11,12]. All these crucial limitations cause the commercial applicability of electrospun fibers to be restrained [13].

Alternatively, centrifugal spinning (CS) is a low-cost process that does not require a high voltage supply, thus holding a high potential for industrial scaling [14,15]. CS is a simple and controllable process that uses centrifugal forces to create fibers, imitating the cotton candy production principle [15,16,17,18,19]. The spinning fluid is placed inside a rotating spinneret. During the rotation of the spinneret, the centrifugal force overcomes the surface tension and viscosity of the spinning fluid to eject a liquid jet from the orifice. Subsequently, the jet is elongated before depositing onto the collector to form solidified micro/nano-fibers. Numerous polymeric materials have been utilized to demonstrate such a CS process, including nylon-6 [20], polyacrylonitrile [21], polycaprolactone [22], polylactic acid [23], and polyvinylpyrrolidone [24]. Yanilmaz et al., prepared a SiO_2_/PAN membrane as a diaphragm material for lithium-ion batteries by the CS method [25]. Nava R et al., prepared a silicon carbide composite fiber based on CS as the non-binder anode for lithium-ion batteries, exhibiting a potential for large-scale production [26]. Some recent studies have focused on CS principles. By optimizing the multiple regression method, Stojanovska et al., investigated the distance between the orifice and collector, the rotation speed, and the diameter of the nozzle to determine the parameters that have a great impact on fiber morphology and diameter [27].

Although ES method can produce fibers with excellent morphology and high distribution uniformity, the low production efficiency and high dependency on polymer properties inhibit its large-scale applications. Despite having high production efficiency, CS tends to produce fibers with morphology and distribution uniformity that are not as good as electrospun fibers [28,29]. Electro-centrifugal spinning (ECS) combines the advantages of ES and CS, which not only greatly expands the application range of CS by producing fibers with good morphology, high uniform distribution, and excellent performance but also dramatically improves the production efficiency [30,31]. A centrifugal drawing force is utilized alongside the electrical force to improve both yield and alignment. Both forces could effectively elongate the polymer jet in ECS to form fibers. In recent years, ECS has been widely used in the production of micro/nano-fibers [32,33]. Based on ECS, Liu et al., prepared uniaxial and cross-oriented ultrafine polystyrene fiber arrays at a lower operating voltage and rotation speed [34]. Khamforoush et al., improved the ECS by employing double nozzles as spinnerets which significantly promoted the production efficiency of micro/nano-fibers [35]. Muller et al., applied ECS to prepare ultra-thin fibers with diameters of tens of nanometers. The productivity of this highly interconnected nano-fiber nonwoven net was several orders of magnitude higher than that of a traditional ES system [30,36]. In order to explore the improvement of the production efficiency of the ECS system, a self-designed electrostatic-field-assisted centrifugal spinning device was used in this work to produce ultrafine PVA fibers. The effect of various process parameters on fiber diameter and morphology was systematically investigated. Importantly, the optimized PVA fiber mat demonstrated the superiority of the highly selective adsorbing performance of ammonia (NH_3_) to CO_2_. The findings presented in this work can be a step forward in producing ultrafine PVA-based fiber materials for many critical applications, such as environmental purification, bioengineering, and NH_3_ storage.

## 2. Materials and Methods

### 2.1. Materials

PVA (polymerization degree: 1700; degree of hydrolysis: 87–89%) was purchased from Aladdin (Shanghai, China). All solutions were prepared in deionized water.

### 2.2. Preparation of Ultrafine PVA Fibers

**Figure 1:**
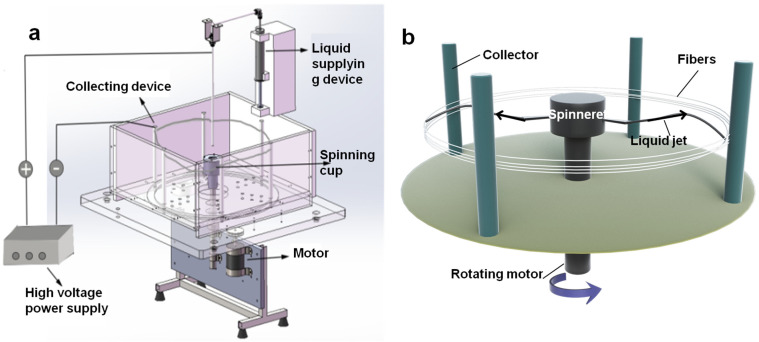
Graphical illustration of the (**a**) ECS setup and the (**b**) formation of PVA fibers.

**Figure 2:**
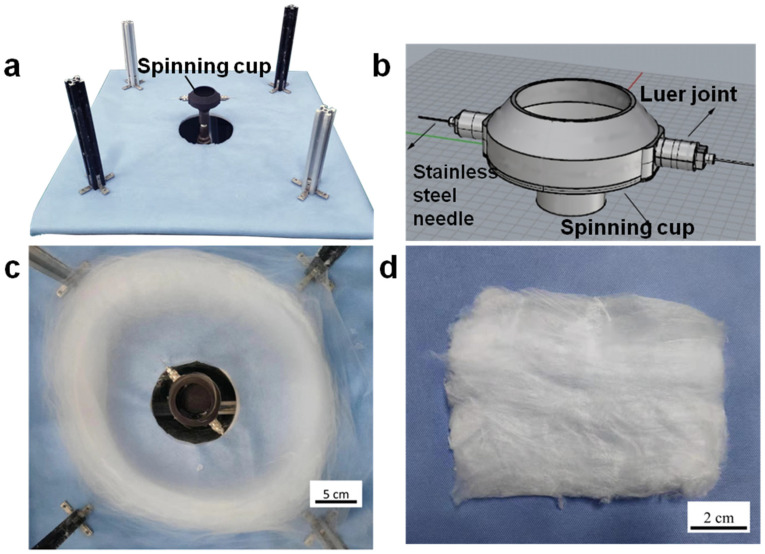
Digital image of the main part of ECS setup (**a**) including a spinning cup with (**b**) two nozzles as the spinneret; (**c**) Digital images of the ECS setup with fibers inside and (**d**) the produced PVA fibers.

In this work, ultrafine fibers were obtained with a very high production efficiency by a self-designed electro-centrifugal spinning (ECS) setup. As shown in Figure 1a, the ECS setup is powered by a motor. The rotation speed of the chamber is controlled in the range from 1000 to 9000 rpm by changing the voltage applied to the motor. A high voltage power supply with a DC voltage range of 8–24 kV is used to supply the electrostatic field. Four metallic cylinders connected to the negative electrode of high voltage supply are employed as the fiber collector (shown in Figure 2a). A metallic tube is connected to the positive electrode. The solutions are added into the chamber through the tube using a syringe pump. The chamber fabricated by the 3D printing technique is connected with two nozzles as the spinneret (Figure 2b). The diameter of the chamber is 6 cm, and the length of the nozzle is 2.5 cm. As the spinning solution, PVA solution with a concentration of 15% was prepared by dissolving PVA powders in distilled water under continuous stirring at 80 °C for 2 h. Then, the obtained solution was kept at room temperature to eliminate bubbles. As Figure 1b shows, during the spinning process, the solution in the chamber was ejected from the nozzle and stretched under the centrifugal and electrostatic forces. The produced fibers were collected onto a collector as shown in Figure 2c. The distance between the center of the spinneret and the collector bar was set to be 30 cm. Because of the low volatile evaporability of water, the temperature and the relative humidity were controlled for all experiments, with temperature above 50 °C and RH below 20% using an additional air conditioning system.

### 2.3. Characterizations

The morphologies of the produced PVA fibers were characterized using a HITACHI S-4800 scanning electron microscope (SEM, Tokyo, Japan). The diameters of the fibers were statistically analyzed using an image processing software (Nano Measurer 1.2) by measuring more than 100 fibers from SEM images for each sample.

### 2.4. Gas Adsorption

The N_2_ adsorption–desorption isotherms (298 K) of the PVA fiber samples were measured using Micromeritics ASAP 2460 adsorption equipment. The BET surface area was calculated from the N_2_ adsorption isotherm data with relative pressures between 0.05 and 0.3 bar at 298 K. The pore size distribution density functional theory (NLDFT) method was derived from the adsorption branch of the N_2_ isotherm by the nonlocal adsorption method. The pore was assumed to be a slit pore model based on the isotherm profile. The total pore volume of the sample was calculated from the amount of N_2_ adsorbed at a relative pressure of 0.99. The micropore area and volume were estimated by the t-plot method.

The ASAP2460 adsorption device and Quantachrome Instrument (version 5.0) were used to determine the adsorption and desorption isotherms of PVA fiber mats for CO_2_ and NH_3_ at 298 K, respectively. Before the gas adsorption measurement, the sample was degassed for approximately 8 h until the weight remained constant.

## 3. Results and Discussion

### 3.1. Morphology Analysis

In this work, in order to obtain ultrafine PVA fibers with a high production efficiency the process parameters were systematically studied and optimized. For the same spinning solution, the centrifugal force plays a critical role in determining the CS fiber morphology, while the ES fiber morphology mainly depends on the electric force. In this ECS spinning system, PVA fibers were formed upon the stretch of the solution by the centrifugal force and the electric force, along with solvent evaporation. Other factors also exist that may affect the fiber morphology and diameter distribution, such as nozzle specification/size, feeding rate, and collection distance. In this work, needles with various inner diameters were employed as nozzles. Primarily, the CS spinning system was used to investigate the effect of a single factor on the PVA fiber morphology, including needle diameter, rotation speed and feeding rate. After the determination of optimum spinning parameters, an electrostatic field was applied to explore the effect of voltage on the spinning performance of the ECS system [14,17,37,38]. In addition, orthogonal experiments were adopted to explore the influence extent of all the parameters mentioned above on the PVA fiber morphology. In the final, ultrafine PVA fibers were successfully produced with an exceptionally high efficiency applying the optimized parameters.

#### 3.1.1. Effect of a Single Factor on the PVA Fiber Morphology

- Nozzle specification

As an important parameter for CS spinneret, the specification of the nozzle affects the solution flow and fiber morphology. The choice of the nozzle specification depends on the polymer of the spinning solution [23,27]. In this work, needles with the inner diameter ranging from 0.3 mm to 0.7 mm were investigated to determine the optimal nozzle specification for the fiber production (Table 1). In the study, the rotation speed of the chamber and the feeding rate of the solution were fixed to be 5500 rpm and 3000 mL h^−1^, respectively.

As predicted, needle 24# with the inner diameter of 0.3 mm was too fine for the high-viscosity PVA solution employed in this experiment to penetrate through without stretching by the electric force. The blocked nozzle becomes incapable of ejecting the liquid jet. As a result, fibers cannot be formed [37]. Conversely, needle 19# with the inner diameter of 0.7 mm was too coarse to stretch the PVA solution into fibers. With the increase in the inner diameter of the needle, the solution droplet in unit time possesses elevating surface area flowing out of the needle under the same feeding rate. The stress distributed over the solution droplet provided by the centrifugal force is decreased with the increase in the droplet surface area [39,40,41]. Consequently, the centrifugal force caused by the rotating chamber under the rotation speed of 5500 rpm was unable to overcome the viscosity and the surface tension of the solution droplet out of needle 19#.

**Figure 3:**
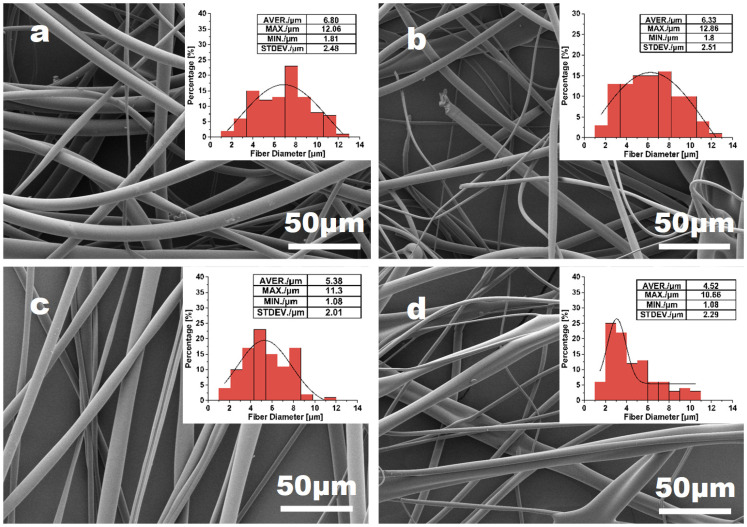
The effect of the nozzle size: (**a**) needle 20# (0.33 mm diameter); (**b**) needle 21# (0.41 mm diameter); (**c**) needle 22# (0.51 mm diameter); (**d**) needle 23# (0.6 mm diameter) on 15% PVA fiber morphology and the diameter distribution, with fixed parameters of rotation speed = 5500 rpm and feeding rate = 3000 mL h^−1^.

**Figure 4:**
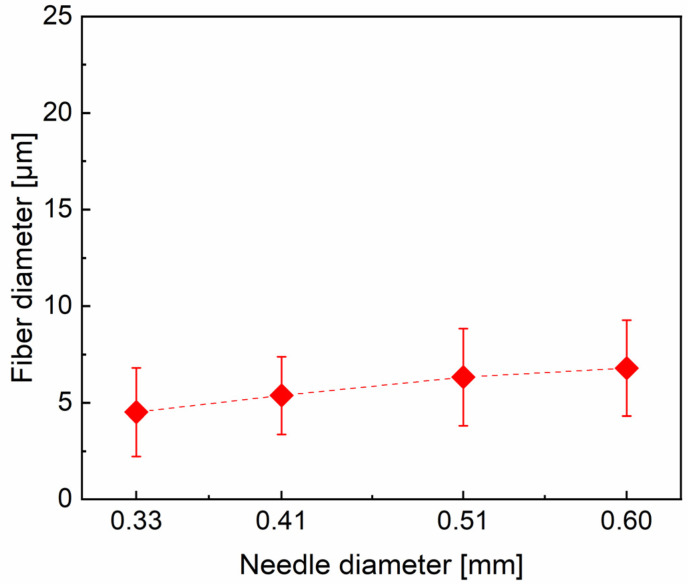
Plot of PVA fiber average diameter as a function of the nozzle size: The inner diameter of 0.33 mm, 0.41 mm, 0.51 mm and 0.6 mm correspond to needle 20#, 21#, 22#, and 23#, respectively.

Figure 3 shows the morphology and diameter distributions of PVA fibers obtained with the nozzle changing from needle 20# to needle 23# (0.60–0.33 mm diameter). The average diameter of PVA fibers is shown in Figure 4. Although the average diameter change in fibers is not significant for all samples, the fiber uniformity varies much more obviously. With needle 20# as the nozzle, the fiber presents a maximum diameter value. As the needle diameter decreases from 0.6 mm to 0.41 mm, the mass of jet in unit time decreases. Consequently, fibers become finer with smoother surface morphology, with the average diameter decreasing from 6.80 μm to 4.52 μm. However, the inner diameter of needle 23# might also be inappropriate for the PVA solution applied in this work. For needle 23#, unsatisfactory spinnability was also evidenced by the unstable spinning process and the wider diameter distribution of the resulting PVA fibers. Overall, based on the analysis of fiber diameter and surface morphology of PVA fiber samples, needle 21# and 22# can be verified to be the optimum choice as the nozzle.

- Rotation speed

As mentioned above, the centrifugal force-enabled stretch of the solution jet during centrifugal spinning can overcome the viscous and surface tension of solution droplet out of the needle tip. The centrifugal force is in direct proportion to the rotation speed of the rotating spinneret. To evaluate the effect of the rotation speed on the spinnability and surface morphology of the produced PVA fibers, experiments at different rotation speeds (3500–7500 rpm) were carried out using the 15% PVA spinning solution and needle 22# with a fixed feeding rate of 3000 mL h^−1^.

**Figure 5:**
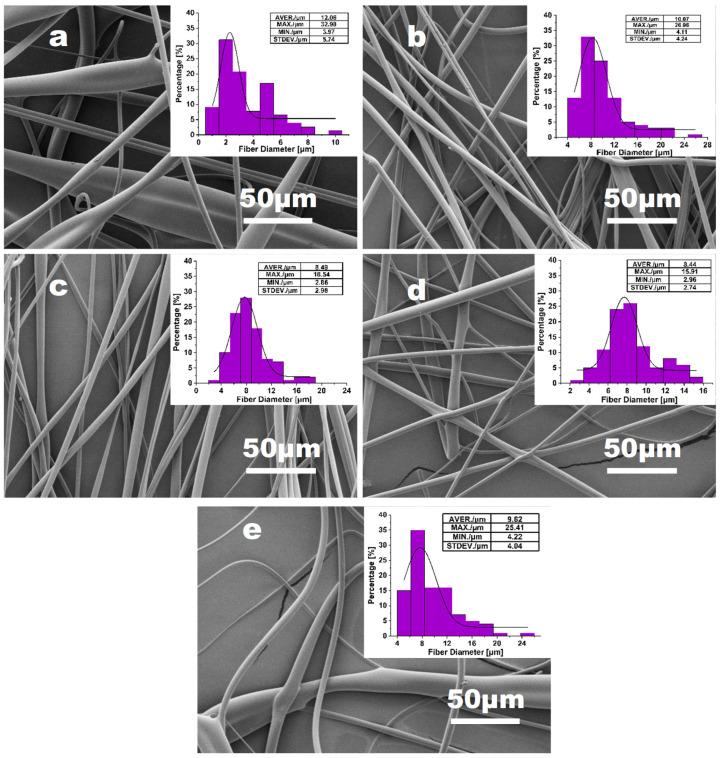
The effect of rotation speed: (**a**) 3500 rpm; (**b**) 4500 rpm; (**c**) 5500 rpm; (**d**) 6500 rpm; (**e**) 7500 rpm on 15% PVA fiber morphology and fiber diameter distribution, with fixed parameters of feeding rate = 3000 mL h^−1^ and needle 22# (0.51 mm diameter).

**Figure 6:**
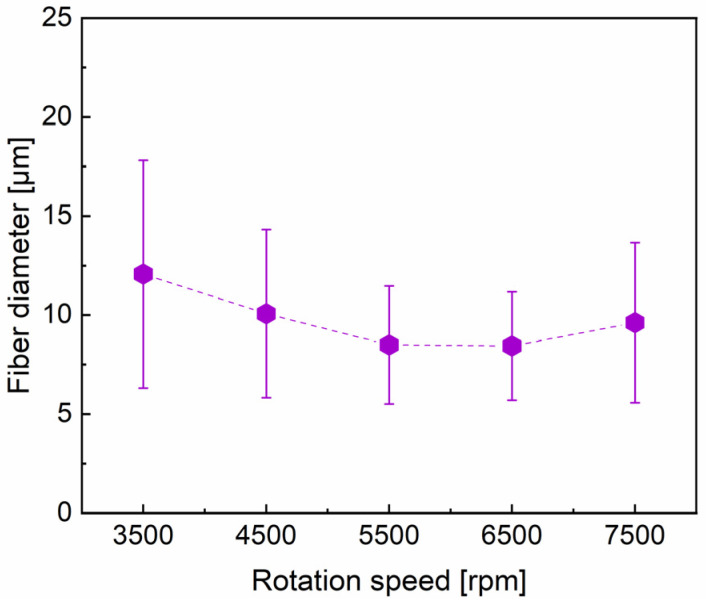
The diameter of PVA fibers prepared under different rotation speeds.

Representative SEM images of the produced fibers and their statistical analysis of fiber diameter distribution are shown in Figure 5 and Figure 6, respectively. An increase in the rotation speed of the spinneret strengthens the centrifugal force and consequently results in the greater elongation of the liquid jet and a decrease in fiber diameters [14,28,42,43]. Notably, the surface morphology and the uniformity of PVA fibers present the tendency to be improved with the decrease in fiber diameter from 12.07 to 8.44 μm, corresponding to a gradual increase in rotation speed from 3500 to 6500 rpm. However, droplets are sprayed rather than being stretched into fibers with the rotation speed larger ≥7500 rpm. A too high rotation speed might result in thick or cracked fibers due to the short flight time and low evaporation of solvent [42]. Figure 5a shows that the liquid jet is not fully elongated into fibers, likely because the centrifugal force produced from the rotational spinneret with the rotation speed of 3500 rpm was insufficient for fiber stretching. As the average fiber diameter decreases, the overall fiber diameter distribution tends to be narrowed [44]. Above all, the best PVA fiber morphology (a fluffy morphology) is obtained at the rotation speed of 6500 rpm, together with a concentrated fiber diameter distribution.

- Effect of feeding rate

**Figure 7:**
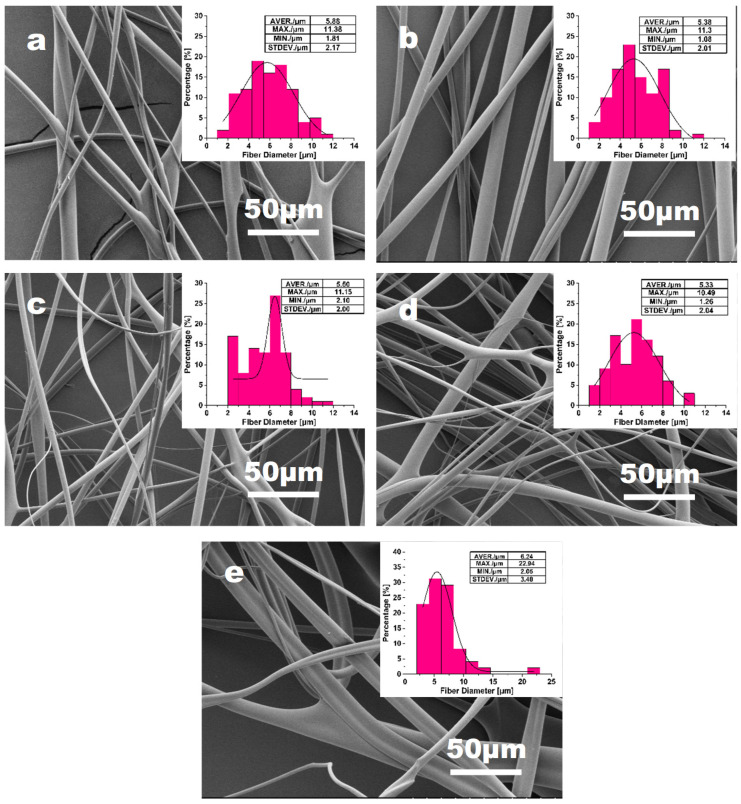
The effect of feeding rate: (**a**) 2900 mL h^−1^; (**b**) 3000 mL h^−1^; (**c**) 3100 mL h^−1^; (**d**) 3200 mL h^−1^; (**e**) 3300 mL h^−1^ on the PVA fiber morphology and fiber diameter distribution, with fixed parameters of rotation speed = 5500 rpm and needle 22# (0.51 mm diameter).

**Figure 8:**
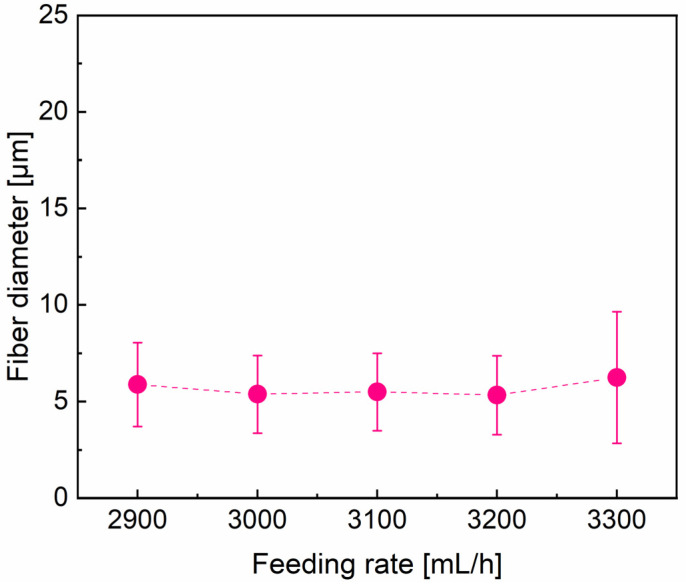
Tendency of average diameter of PVA fibers with the increase in feeding rate.

The variations of fiber morphology and diameter distribution of the PVA fibers with varying feeding rates (ranging from 2900 to 3300 mL h^−1^) are presented in Figure 7 and Figure 8. To establish the correlation between fiber morphology and feeding rates, the solution concentration, nozzle size, and rotation speed were fixed to be 15%, 22#, and 5500 rpm, respectively. Figure 8 shows that there is no significant correlation between the average fiber diameter and the feeding rates. However, the fiber morphology and the diameter distribution show that fibers would display obvious inhomogeneity if the feeding rate goes beyond a critical value (approximately 3100 mL h^−1^). The feeding rate with higher than 3100 mL h^−1^ would cause a rapid accumulation of the solution at the needle tip. Consequently, the solution streams could not be elongated adequately into fibers because of the lower shearing force and tensile force on the surface per unit volume solution [45]. Furthermore, there would not be enough time for water to be evaporated if there is too much aqueous solution, which is another crucial reason for the formation of heterogeneous fiber [37]. However, a feeding rate that is too low can generate another issue. The high velocity airflow surrounding the spinneret causes the solution jet losing its solvent rapidly. As a consequence, the extension of the jet becomes more difficult, resulting in the formation of thicker fibers with a wider diameter distribution, which is not favorable for the production of ultrafine PVA fibers.

- Effect of the electrostatic field voltage

**Figure 9:**
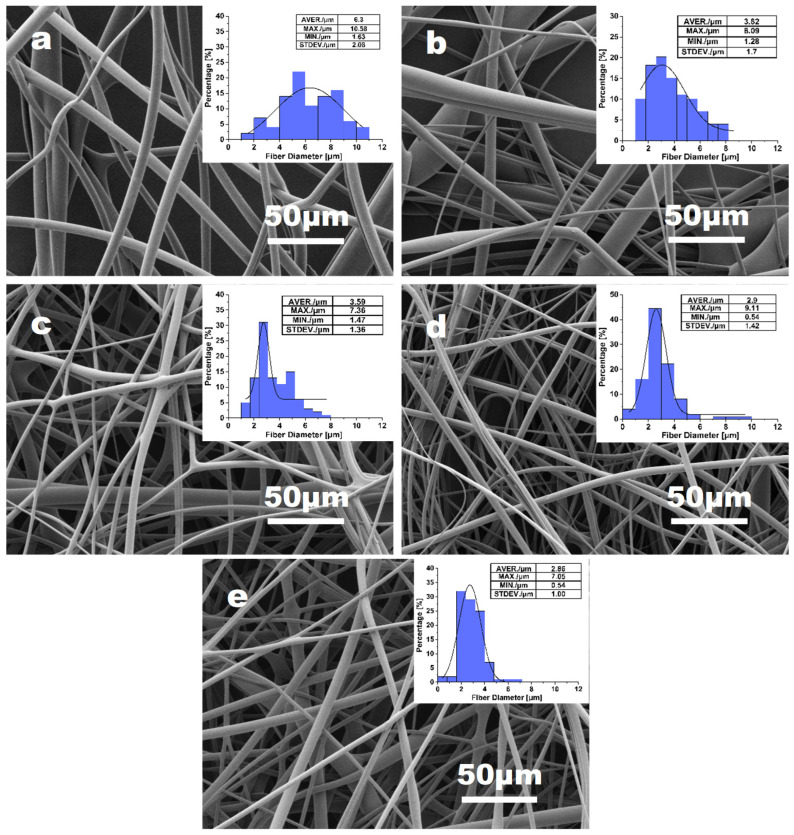
The effect of different voltages: (**a**) 8 kV; (**b**) 12 kV; (**c**) 16 kV; (**d**) 20 kV; (**e**) 24 kV on 15% PVA fiber morphology and fiber diameter distribution, with fixed parameters of 22# (0.51 mm diameter) nozzle size, rotation speed = 5500 rpm and feeding rate = 3000 mL h^−1^.

**Figure 10:**
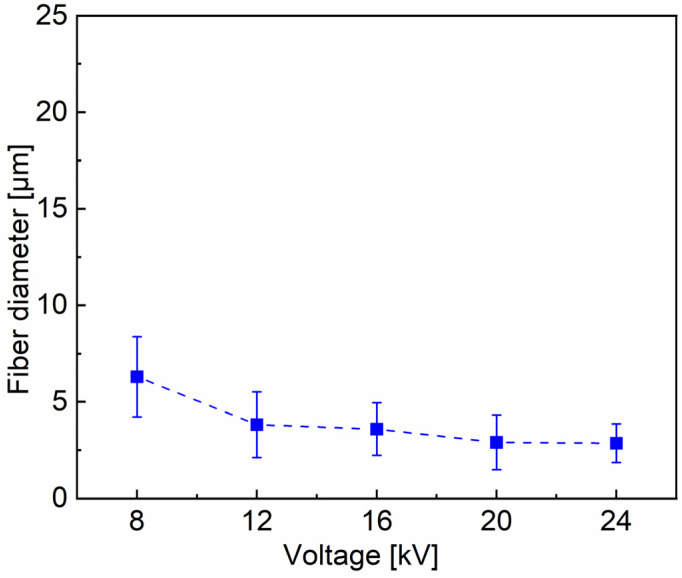
Tendency of average diameter of PVA fibers with applied voltage.

For obtaining uniform PVA fibers controlling the stability and the stretching of the spinning fluid jet is essential in centrifugal spinning. As proved by previous explorations on electrospinning technique, the electrostatic force is an extremely efficient dragline force in solution jet stretching [46]. Therefore, the CS device is further promoted to establish the electrostatic-assisted centrifugal spinning setup. Introducing an additional electrostatic field into the CS system can, to a certain extent, overcome the problem of unstable liquid jet. The jets with a positive charge repel each other upon the applied voltage, while the collector with a negative charge provides an attraction force to the jets. As a consequence, the combination of electric field force and centrifugal force can easily overcome the surface tension of the solution droplet and elongate solution jet into much thinner fibers. A series of experiments are performed by altering the applied voltage in the range of 8 to 24 kV, with the nozzle size, rotation speed, and feeding rate fixed to be 22#, 5500 rpm, and 3000 mL h^−1^, respectively (Figure 9). The average fiber diameter decreases from 6.30 to 2.86 μm with an increase in the applied voltage from 8 to 24 kV (Figure 10). It is obvious that the introduction of an electrostatic field made an exceeding improvement on the spinning stability and the fiber morphology when compared with CS technology. The fibers produced using ECS setup present much smaller diameter and narrower diameter distribution. An increase in the applied voltage definitely generates an increased electrostatic field force, thereby inducing greater stretching force on the solution jet. As a result, fibers get more sufficiently elongated to be thinner and more homogeneous with the increase in voltage. The studies indicate that the applied voltage strongly affects the fiber alignment. The applying low voltage of 8 kV enables a mass of fibers to be stuck together. While with a high voltage of 24 kV, the instability of the jet worsens the fiber uniformity. At the 20 kV voltage the fiber distribution becomes highly homogeneous (Figure 9d).

#### 3.1.2. Orthogonal Experimental Design of Centrifugal Spinning

**Figure 11:**
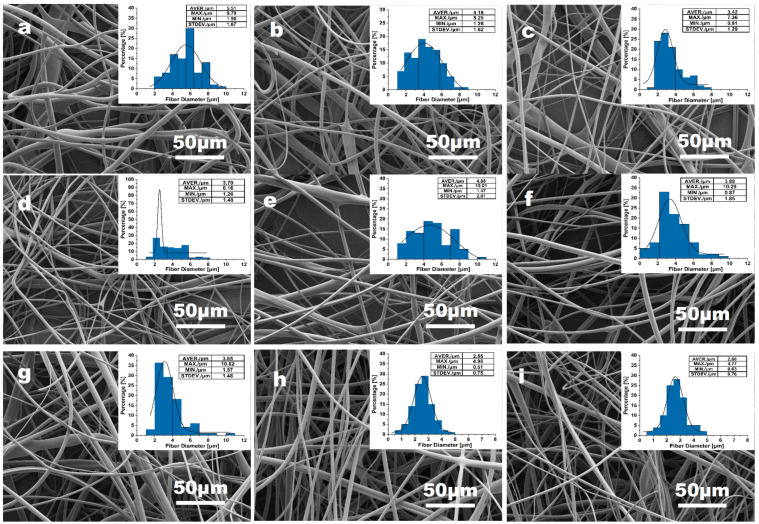
PVA fibers obtained from the orthogonal experiment of (**a**) experiment No. 1; (**b**) experiment No. 2; (**c**) experiment No. 3; (**d**) experiment No. 4, (**e**) experiment No. 5, (**f**) experiment No. 6; (**g**) experiment No. 7; (**h**) experiment No. 8; (**i**) experiment No. 9.

According to the single-factor experiments, value ranges of the four above parameters appropriate for the PVA fiber spinning were determined. To obtain ultrafine fibers and further optimize the centrifugal spinning parameters, the L9 (39) orthogonal test was performed to investigate the impact of four factors including needle size (factor A), feeding speed (factor B), rotation speed (factor C), and electrostatic field voltage (factor D) on the fiber morphology. Average diameter and standard deviation of the average diameter are the evaluation indices of the orthogonal experiment in the study. The three levels of the four factors are shown in Table 2, and the analysis results of the orthogonal test are given in Table 3 and Table 4. The SEM study results and PVA fiber diameter distribution are presented in Figure 11.

Figure 11 shows that PVA fibers could be possibly obtained by all planned experiments. A range analysis table is provided for the nine group experiments (experiment No. 1–9 in Table 3 and Table 4). Among the nine group experiments, experiment No. 8 presents fiber samples with the best surface morphology (Figure 11h), an average diameter of 2.55 μm, and the narrowest fiber distribution. For a further analysis of the results, there are two indexes in the two tables (Table 3 and Table 4). K values represent the mean values of average diameters and standard deviation of diameters for A–D factors at various levels. Thus, in order to get ultrafine PVA fibers the value of K should be as small as possible. According to the value of the index (KA1 > KA2 > KA3, KB1 > KB2 > KB3, KC3 > KC1 > KC2, KD1 > KD2 > KD3) presented in Table 3, the optimum parameters of rotation speed, nozzle size, feeding rate and voltage for ultrafine fibers formation can be 6500 rpm, 0.41 mm, 3100 mL h^−1^, and 20 kV, respectively. R values reflect the effect of a conducive or detrimental level on the average diameter and its standard deviation. The maximum R value corresponds to the most important factor. By comparing the two sets of R values, it is obvious that the influences of rotation speed of the spinneret and electrostatic filed voltage are much higher than the other two factors. Simultaneously, both the feeding rate and the needle specification could exert impacts on the fiber morphology.

In this work, ultrafine PVA fibers (average diameter of 2.55 μm) with the minimum diameter of roughly 510 nm were successfully produced with an exceptional high efficiency (3000 mL h^−1^) by the self-designed ECS system. The PVA fibers produced are not as fine as PVA fibers generated by the ES system and CS system in the literature [47], which can be related with the much higher CS rotation speed, higher ES voltage, and almost twice larger PVA molecules employed in the work [43]. Therefore, the PVA fibers produced by the self-designed ECS system can be possibly further optimized [47,48].

Significantly, the PVA fiber production efficiency of this self-designed ECS system is about 1000 times higher than that of ES system. The feeding rate in ECS process reaches up to 3000 mL h^−1^, while usually the feeding rate in the traditional ES process can be as low as just a few milliliters per hour [9,12,13,14,15]. Thus, ultrafine PVA fibers can be potentially produced by the ECS system on a large scale.

### 3.2. Adsorption Performance toward NH_3_ and CO_2_

**Figure 12:**
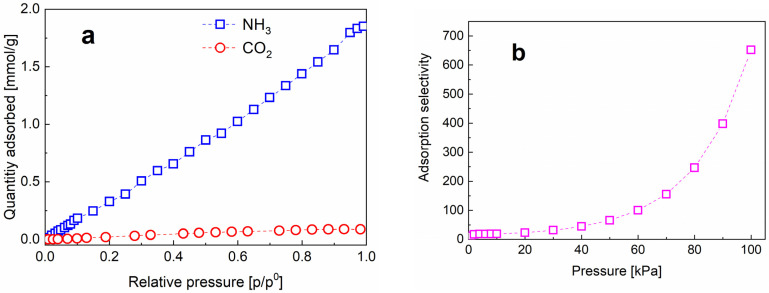
(**a**) The adsorptive performance toward two gases of NH_3_ and CO_2_ and (**b**) the adsorption selectivity of NH_3_/CO_2_.

The air pollution caused by NH_3_ leakage from the NH_3_ chemical industry is becoming more severe since NH_3_ is irreplaceable in the fields of fertilizers and pharmaceutical products. Various adsorbents have been investigated for the removal of NH_3_ leakage. In addition, as a sustainable fuel, NH_3_ has become more promising in the fuel cell industry, thereby stimulating the material researches for NH_3_ storage [49,50]. Due to the multiple hydroxyl groups of PVA molecules which can interact with NH_3_ molecules, PVA ultrafine fibers would be a good candidate as the NH_3_ adsorbent [49]. To demonstrate the viable application of the best prepared PVA fibers (experiment No. 8) from the above experiments, their adsorptive performance toward two gases NH_3_ and CO_2_ of great concern was investigated. The single-component isotherms of NH_3_ and CO_2_ over the ultrafine PVA fiber mats were measured at 298 K, as shown in Figure 12. As presumed, the cotton-like PVA fiber mats present a capacity of adsorbing NH_3_. NH_3_ molecules can be adsorbed via constructing hydrogen bonds with hydroxyl groups of PVA molecules on the surface of fibers, which make a contribution to inducing the gate opening at relatively low pressures [49]. Although the obtained PVA fibers present lower adsorption capability than MOF (Metal-organic framework) materials for NH_3_ adsorption [50], the much lower price of PVA products is still very competitive. In addition, the NH_3_ adsorption performance of PVA fibers can be further improved by the introduction of hierarchically porous structures [11,51]. The optimal PVA fiber mats exhibit adsorption selectivity to NH_3_ over CO_2_ (shown in Figure 12b) [52], and limited adsorption capacity can be noted for CO_2_ in stark contrast to the decent adsorption capacity toward NH_3_. Therefore, a useful alternative to traditional adsorptive materials (such as activated carbon) is provided to capture the hazardous NH_3_ for purifying the living environment around NH_3_ producing factories and also for NH_3_ storage application. In addition, the present PVA fiber mates produced using the novel self-designed ECS setup is expected to be extended to other PVA-based functional materials for many critical applications even beyond environmental purification such as flexible sensors and electronics, and NH_3_ storage applications.

## 4. Conclusions

In this work, ultrafine PVA fibers were successfully produced with high efficiency by a self-designed electrostatic-field-assisted centrifugal spinning system. Compared with traditional centrifugal spinning systems, the present approach based on the spinning apparatus by introducing an additional electrostatic field can efficiently produce ultrafine fibers. Meanwhile, the PVA fiber production efficiency of the ECS system (≥3000 mL h^−1^) is around 1000 times higher than that of a traditional electrospinning system (around few milliliters per hour). For obtaining ultrafine PVA fibers by the designed ECS system, the effects of rotation speed, needle size, feeding rate, and voltage on fiber morphology were systematically investigated. The orthogonal test was used to explore the optimum parameters. The rotation speed was demonstrated to be the most critical parameter for the production of PVA fibers by ECS system. Ultrafine PVA fibers with the diameter of 2.55 μm were successfully obtained applying the spinning parameters, including rotation speed (6500 rpm), needle size (0.51 mm), feeding rate (3000 mL h^−1^), and voltage (20 kV). The optimal PVA fiber mat was demonstrated to be capable of selectively capturing NH_3_ relative to CO_2_. Therefore, this work opens up a new avenue for producing ultrafine fiber materials for many critical applications beyond environmental purification, such as flexible sensors and electronics, and NH_3_ storage applications.

## Figures and Tables

**Table 1:** The diameter of needles.

Needle Number	Inner Diameter [mm]
19#	0.70
20#	0.60
21#	0.51
22#	0.41
23#	0.33
24#	0.3

**Table 2:** Orthogonal test factor levels which are applied in Table 3 and Table 4.

Test Level	Rotation Speed [rpm] (Factor A)	Nozzle Size [mm] (Factor B)	Feeding Rate [mL/h] (Factor C)	Voltage [kV] (Factor D)
I	4500	0.60	3000	+6, −6
II	5500	0.51	3100	+8, −8
III	6500	0.41	3200	+10, −10

**Table 3:** Orthogonal test analysis on the average diameter of ECS fibers.

Experiment No.	Rotation Speed (Factor A)	Nozzle Size (Factor B)	Feeding Rate (Factor C)	Voltage (Factor D)	Average Diameter of ECS Fibers [μm]
**1**	I	I	I	I	5.51
**2**	I	II	II	II	4.16
**3**	I	III	III	III	3.42
**4**	II	I	II	III	3.79
**5**	II	II	III	I	4.88
**6**	II	III	I	II	3.88
**7**	III	I	III	II	3.65
**8**	III	II	I	III	2.55
**9**	III	III	II	I	2.68
**K1**	4.363	4.317	3.980	4.357	T = 34.52
**K2**	4.183	3.863	3.543	3.897	
**K3**	2.960	3.327	3.983	3.253	
**R**	1.403	0.990	0.440	1.104	
**Order of importance**	A > D > B > C				
**Optimal level**	A3	B3	C2	D3	

K = ∑the value of evaluation indexes at the same level of each factor/3; R = max {K}–min{K}.

**Table 4:** Orthogonal test analysis on the diameter standard deviation of ECS fibers.

Experiment No.	Rotation Speed (Factor A)	Nozzle Size (Factor B)	Feeding Rate (Factor C)	Voltage (Factor D)	Standard Deviation of Average Diameter [μm]
**1**	I	I	I	I	1.67
**2**	I	II	II	II	1.62
**3**	I	III	III	III	1.29
**4**	II	I	II	III	1.48
**5**	II	II	III	I	2.01
**6**	II	III	I	II	1.85
**7**	III	I	III	II	1.48
**8**	III	II	I	III	0.75
**9**	III	III	II	I	1.67
**K1**	1.53	1.54	1.42	1.48	T = 12.91
**K2**	1.78	1.46	1.29	1.65	
**K3**	1.00	1.3	1.59	1.17	
**R**	0.78	0.24	0.31	0.48	
**Order of importance**	A > D > C > B				
**Optimal level**	A3	B3	C2	D3	

K = ∑the value of evaluation indexes at the same level of each factor/3; R = max {K}–min{K}.

## Data Availability

The data presented in this study are available on request from the corresponding author.

## References

[1] Kuk E., Ha Y.M., Yu J., Im I.T., Kim Y., Jung Y.C. (2016). Robust and Flexible Polyurethane Composite Nanofibers Incorporating Multi-Walled Carbon Nanotubes Produced by Solution Blow Spinning. Macromol. Mater. Eng..

[2] Han X.W., Zhang H.W., Luo H.Y., Zheng X.L., Yang Z., Hu N. (2018). Preparation of Poly (vinyl alcohol) Microspheres Based on Droplet Microfluidic Technology. Chin. J. Anal. Chem..

[3] Nayak R., Padhye R., Kyratzis I.L., Truong Y.B., Arnold L. (2012). Recent advances in nanofibre fabrication techniques. Text. Res. J..

[4] Feng L., Li S., Li H., Zhai J., Song Y., Jiang L., Zhu D. (2002). Super-Hydrophobic Surface of Aligned Polyacrylonitrile Nanofibers. Angew. Chem..

[5] Ondarçuhu T., Joachim C. (1998). Drawing a single nanofibre over hundreds of microns. Europhys. Lett..

[6] Simonet M., Schneider O.D., Neuenschwander P., Stark W.J. (2007). Ultraporous 3D polymer meshes by low-temperature electrospinning: Use of ice crystals as a removable void template. Polym. Eng. Sci..

[7] Son W.K., Youk J.H., Lee T.S., Park W.H. (2004). The effects of solution properties and polyelectrolyte on electrospinning of ultrafine poly(ethylene oxide) fibers. Polymer..

[8] Luo C.J., Nangrejo M., Edirisinghe M. (2010). A novel method of selecting solvents for polymer electrospinning. Polymer..

[9] Greiner A., Wendorff J.H. (2007). Electrospinning: A fascinating method for the preparation of ultrathin fibers. Angew. Chem. Int. Ed. Engl..

[10] Hassan M., Ibrahim, Klingner A. (2020). A review on electrospun polymeric nanofibers: Production parameters and potential applications. Polym. Test..

[11] Jiang C., Tian Y., Wang L., Zhao S., Hua M., Yao L. (2023). Facile Approach for the Potential Large-Scale Production of Polylactide Nanofiber Membranes with Enhanced Hydrophilic Properties. Materials.

[12] Tutak W., Sarkar S., Lin-Gibson S., Farooque T.M., Jyotsnendu G., Wang D. (2013). The support of bone marrow stromal cell differentiation by airbrushed nanofiber scaffolds. Biomaterials.

[13] Luo C.J., Stoyanov S.D., Stride E., Pelan E., Edirisinghe M. (2012). Electrospinning versus fibre production methods: From specifics to technological convergence. Chem. Soc. Rev..

[14] Badrossamay M.R., McIlwee H.A., Goss J.A., Parker K.K. (2010). Nanofiber assembly by rotary jet-spinning. Nano Lett..

[15] Ren L., Ozisik R., Kotha S.P., Underhill P.T. (2015). Highly Efficient Fabrication of Polymer Nanofiber Assembly by Centrifugal Jet Spinning: Process and Characterization. Macromolecules.

[16] Atıcı B., Ünlü C.H., Yanilmaz M. (2022). A Review on Centrifugally Spun Fibers and Their Applications. Polym. Rev..

[17] Stojanovska E., Canbay E., Pampal E.S., Calisir M.D., Agma O., Polat Y. (2016). A review on non-electro nanofibre spinning techniques. RSC Adv..

[18] Amalorpava Mary L., Senthilram T., Suganya S., Nagarajan L., Venugopal J., Ramakrishna S., Giri Dev V.R. (2013). Centrifugal spun ultrafine fibrous web as a potential drug delivery vehicle. Express Polym. Lett..

[19] Vanheusden C., Vanminsel J., Reddy N., Samyn P., D’Haen J., Peeters R., Ethirajan A., Buntinx M. (2023). Fabrication of poly(3-hydroxybutyrate-co-3-hydroxyhexanoate) Fibers Using Centrifugal Fiber Spinning: Structure, Properties and Application Potential. Polymers.

[20] Hammami M.A., Krifa M., Harzallah O. (2014). Centrifugal force spinning of PA6 nanofibers—Processability and morphology of solution-spun fibers. J. Text. Inst..

[21] Dabirian F., Hosseini Ravandi S., Pishevar A. (2010). Investigation of Parameters Affecting PAN Nanofiber Production Using Electrical and Centrifugal Forces as a Novel Method. Curr. Nanosci..

[22] Zander N.E. (2014). Formation of melt and solution spun polycaprolactone fibers by centrifugal spinning. J. Appl. Polym. Sci..

[23] O’Haire T., Rigout M., Russell S.J., Carr C.M. (2014). Influence of nanotube dispersion and spinning conditions on nanofibre nanocomposites of polypropylene and multi-walled carbon nanotubes produced through Forcespinning TM. J. Thermoplast. Compos..

[24] Ren L., Pandit V., Elkin J., Denman T., Cooper J.A., Kotha S.P. (2013). Large-scale and highly efficient synthesis of micro- and nano-fibers with controlled fiber morphology by centrifugal jet spinning for tissue regeneration. Nanoscale.

[25] Yanilmaz M., Lu Y., Li Y., Zhang X. (2015). SiO_2_/polyacrylonitrile membranes via centrifugal spinning as a separator for Li-ion batteries. J. Power Sources.

[26] Nava R., Cremar L., Agubra V., Sánchez J., Alcoutlabi M., Lozano K. (2016). Centrifugal Spinning: An Alternative for Large Scale Production of Silicon-Carbon Composite Nanofibers for Lithium Ion Battery Anodes. ACS Appl. Mater. Inter..

[27] Stojanovska E., Kurtulus M., Abdelgawad A., Candan Z., Kilic A. (2018). Developing lignin-based bio-nanofibers by centrifugal spinning technique. Int. J. Biol. Macromol..

[28] Sun J., Zhang Z., Lu B., Mei S., Xu Q., Liu F. (2018). Research on parametric model for polycaprolactone nanofiber produced by centrifugal spinning. J. Braz. Soc. Mech. Sci. Eng..

[29] Li Z., Mei S., Dong Y., She F., Kong L. (2019). High Efficiency Fabrication of Chitosan Composite Nanofibers with Uniform Morphology via Centrifugal Spinning. Polymers.

[30] Gu J., Yagi S., Meng J., Dong Y., Qian C., Zhao D., Kumar A., Xu T., Lucchetti A., Xu H. (2022). High-efficiency production of core-sheath nanofiber membrane via co-axial electro-centrifugal spinning for controlled drug release. J. Membr. Sci..

[31] Xu H., Yagi S., Ashour S., Du L., Hoque M.E., Tan L. (2023). A Review on Current Nanofiber Technologies: Electrospinning, Centrifugal Spinning, and Electro-Centrifugal Spinning. Macromol. Mater. Eng..

[32] Arhaj S., Conway B.R., Ghori M.U. (2023). Nanofibres in Drug Delivery Applications. Fibers.

[33] Su X., Jia C., Xiang H., Zhu M. (2023). Research progress in preparation, properties, and applications of medical protective fiber materials. Appl. Mater. Today.

[34] Liu S.-L., Long Y.-Z., Zhang Z.-H., Zhang H.-D., Sun B., Zhang J.-C., Han W.-P. (2013). Assembly of Oriented Ultrafine Polymer Fibers by Centrifugal Electrospinning. J. Nanomater..

[35] Khamforoush M., Asgari T. (2015). A Modified Electro-Centrifugal Spinning Method to Enhance the Production Rate of Highly Aligned Nanofiber. Nano.

[36] Müller F., Jokisch S., Bargel H., Scheibel T. (2020). Centrifugal Electrospinning Enables the Production of Meshes of Ultrathin Polymer Fibers. ACS Appl. Polym. Mater..

[37] Lu Y., Li Y., Zhang S., Xu G., Fu K., Lee H., Zhang X. (2013). Parameter study and characterization for polyacrylonitrile nanofibers fabricated via centrifugal spinning process. Eur. Polym. J..

[38] Yang S.B., Yeum J.H. (2017). Morphological Comparison of Aligned Poly(vinyl alcohol) Nanofibers Fabricated by Modified Electrospinning and Centrifugal Jet Spinning Techniques. J. Nanosci. Nanotechnol..

[39] Taghavi S.M., Larson R.G. (2014). Erratum: Regularized thin-fiber model for nanofiber formation by centrifugal spinning. Phys. Rev. E.

[40] Ren L., Kotha S.P. (2014). Centrifugal Jet Spinning for Highly Efficient and Large-scale Fabrication of Barium Titanate Nanofibers. Mater Lett..

[41] Zhang Z., Sun J. (2017). Research on the development of the centrifugal spinning. MATEC Web Conf..

[42] Zhang X., Lu Y. (2014). Centrifugal Spinning: An Alternative Approach to Fabricate Nanofibers at High Speed and Low Cost. Polymer Rev..

[43] Zhiming Z., Boya C., Zilong L., Jiawei W., Yaoshuai D. (2020). Spinning solution flow model in the nozzle and experimental study of nanofibers fabrication via high speed centrifugal spinning. Polymer.

[44] Weng B., Xu F., Salinas A., Lozano K. (2014). Mass production of carbon nanotube reinforced poly(methyl methacrylate) nonwoven nanofiber mats. Carbon.

[45] Medeiros E.S., Glenn G.M., Klamczynski A.P., Orts W.J., Mattoso L.H.C. (2009). Solution blow spinning: A new method to produce micro- and nanofibers from polymer solutions. J. Appl. Polymer. Sci..

[46] Tang D., Zhuang X., Zhang C., Cheng B., Li X. (2015). Generation of nanofibers via electrostatic-Induction-assisted solution blow spinning. J. Appl. Polymer. Sci..

[47] Rihova M., Ince A.E., Cicmancova V., Hromadko L., Castkova K., Pavlinak D. (2021). Water-born 3D nanofiber mats using cost-effective centrifugal spinning: Comparison with electrospinning process: A complex study. J. Appl. Polym. Sci..

[48] Gholipour-Kanani A., Daneshi P. (2022). A Review on Centrifugal and Electro-Centrifugal Spinning as New Methods of Nanofibers Fabrication. J. Text. Polym..

[49] Kang D.W., Kang M., Kim H., Choe J.H., Kim D.W., Park J.R. (2019). A Hydrogen-Bonded Organic Framework (HOF) with Type IV NH_3_ Adsorption Behavior. Angew. Chem..

[50] Shen C., Wang P., Shen L., Yin X., Miao Z. (2022). NH_3_ Adsorption Performance of Silicon-Supported Metal Chlorides. Ind. Eng. Chem. Res..

[51] Roberts A.D., Lee J.M., Magaz A., Smith M.W., Dennis M., Scrutton N.S., Blaker J.J. (2020). Hierarchically Porous Silk/Activated-Carbon Composite Fibres for Adsorption and Repellence of Volatile Organic Compounds. Molecules.

[52] Liu Z., Wu Y., Liu B., Oh S.C., Fan W., Qian Y., Xi H. (2016). Tuning the adsorption and separation properties of noble gases and N_2_ in CuBTC by ligand functionalization. RSC Adv..

